# All-Cause Death and Major Adverse Events in Atrial Fibrillation with Frailty: Observations from the Korea National Health Insurance Service Data

**DOI:** 10.31083/j.rcm2502052

**Published:** 2024-01-30

**Authors:** Jong Sung Park, Pil-Sung Yang, Daehoon Kim, Jung-Hoon Sung, Eunsun Jang, Hee Tae Yu, Tae-Hoon Kim, Hui-Nam Pak, Moon-Hyoung Lee, Boyoung Joung

**Affiliations:** ^1^Division of Cardiology, Department of Internal Medicine, Severance Cardiovascular Hospital, Yonsei University College of Medicine, 03722 Seoul, Republic of Korea; ^2^Department of Cardiology, CHA Bundang Medical Center, CHA University, 13496 Seongnam, Republic of Korea

**Keywords:** atrial fibrillation, frailty, outcomes, anticoagulation, bleeding

## Abstract

**Background:**

Atrial fibrillation (AF) is an indicator of frailty in old patients. This study aimed to investigate the effect of frailty on the use of oral anticoagulants (OAC) and clinical outcomes in a nationwide cohort of patients with new-onset AF.

**Methods:**

This study included 451,368 participants without AF from the Korea National Health Insurance Service-Health Screening cohort between 2002 and 2009. The Hospital Frailty Risk Score was retrospectively calculated for each patient using all available International Classification of Disease 10th revision diagnostic codes. According to the aggregate score, patients were divided into two groups: the participants without frailty (<5 points) and the participants with frailty (≥5 points). The primary outcome was death from any cause, and the secondary outcomes were cardiovascular death, ischemic stroke, major bleeding, and heart failure admission.

**Results:**

With up to 7.2 ± 1.5 years of follow-up, 11,953 participants (median age, 67 [interquartile range, 59.5–74.5] years; 7200 [60.2%] males) developed new-onset AF. Among the patients with AF, 3224 (26.9%) had frailty. Frailty was significantly associated with old age, female sex, polypharmacy, and other comorbidities. In patients with AF, frailty was negatively associated with OAC prescription after new-onset AF (*p* < 0.001). Compared to patients without frailty, patients with frailty had a significantly higher incidence and risk of all-cause death (hazard ratio [HR] 2.88, 95% confidence interval [CI] 2.65–3.14), cardiovascular death (HR 2.42, 95% CI 2.10–2.80), ischemic stroke (HR 2.25, 95% CI 2.02–2.51), major bleeding (HR 2.44, 95% CI 2.17–2.73), and heart failure admission (HR 1.29, 95% CI 1.09–1.52). In subgroup analysis, when compared to the non-OAC group, the risks associated with frailty were significantly lower in the OAC group for all-cause death, cardiovascular death, ischemic stroke, and heart failure admission.

**Conclusions:**

Frailty was negatively associated with the use of OAC and was a predictor of poor prognosis owing to the association of frailty with death, thromboembolic events, bleeding, and heart failure admission. However, OAC use was associated with lower risks related to frailty for all-cause death and major adverse cardiovascular events in patients with AF.

## 1. Introduction

Atrial fibrillation (AF) is a prevalent form of supraventricular tachyarrhythmia. Furthermore, AF is associated with an elevated risk of both mortality and morbidity resulting from ischemic events, stroke, and aggravation of heart failure, resulting in a high burden of healthcare costs [1, 2, 3, 4, 5]. The worldwide AF epidemic is mainly attributed to an increasingly aging population [6]. Frailty is a state of reduced physiological reserves and stress resistance. Moreover, frailty has been recognized as an important factor associated with adverse clinical outcomes among old patients as a result of the progressive deterioration of various physiological systems, diminished homeostatic reserve, and reduced resilience [7, 8]. Fumagalli *et al*. [9] reported that AF could be a marker of frailty, especially in old patients, and Marzona *et al*. [10] have reported a loss of independence in performing activities of daily living in a follow-up of aged patients with AF. Patients with AF may exhibit a four-fold increased odds ratio for frailty compared to patients without AF [11]. Therefore, careful consideration is needed when determining the treatment for frail aged patients with AF.

As the optimal approach for managing AF in older patients with frailty remains uncertain, guidelines and consensus statements suggest the adoption of a personalized and patient-centered strategy [12]. For example, catheter ablation has demonstrated superiority over antiarrhythmic medication in maintaining sinus rhythm and enhancing the quality of life in patients with AF [13, 14]. However, in frail older patients with AF, this approach may not be helpful because of the high morbidity and mortality. Similarly, the optimal medical treatment for AF in a frail population may differ from that in a non-frail population in terms of polypharmacy, a variety of medications, and doses of medication [15, 16, 17]. According to a recent study, ablation is potentially associated with a decreased risk of mortality and composite outcomes in non-frail older patients with AF. However, no substantial advantageous impact of ablation was observed in older individuals with frailty who were diagnosed with AF [15]. Further studies can help provide a clear understanding of the effectiveness of ablation in different patient populations, including frail individuals with AF. Although the degree of benefit decreased as frailty increased, the advantage of implementing early rhythm control strategies in managing AF concerning cardiovascular outcomes was consistent, without an elevated risk of adverse outcomes [17]. Among frail patients with AF, oral anticoagulant (OAC) treatment has been associated with favorable clinical outcomes. Among frail patients with AF, OACs are associated with reduced incidence of ischemic stroke, bleeding, and mortality [16]. Many patients with AF and frailty could not be prescribed AF medications or optimal doses of medications due to their high bleeding risk and poor general condition in the real medical field. This gap between the ideal and the real treatment direction may have an important effect on the clinical outcomes of aged patients with AF and frailty. Hence, merit is present in investigating this gap and the clinical outcomes using real-world data.

Previous evidence suggests that frail patients with AF are more likely to experience adverse events [15, 16, 17]. Therefore, frailty is important for estimating risks and aiding in the diagnosis and care planning of older patients with AF [18]. However, the prevalence of frailty, its association with treatment, and its impact on the outcomes of patients with AF have not been well elucidated. The objectives of this study were to examine (i) the prevalence of frailty and its potential association with OAC and anti-platelet agents, (ii) the impact of frailty on clinical outcomes, and (iii) the impact of OAC use on clinical outcomes in patients with AF, with or without frailty.

## 2. Materials and Methods

### 2.1 Data Source

The present study utilized the Korea National Health Insurance Service-Health Screening (NHIS-HealS) cohort released in 2015, which has been previously characterized in detail [19, 20]. The cohort comprises 514,764 Korean individuals aged from 40 to 80 years, who were initially enrolled in 2002 and were subsequently followed up until 2013, with pertinent data on lifestyle and behaviors obtained through questionnaires, as well as major findings of health examinations. In Korea, most people are enrolled in a single national healthcare insurance system provided by the government. Every insured adult is entitled to participate in a comprehensive health screening program that takes place every 2 years. The study cohort was drawn from a random sample of 10% of health-screening participants who underwent screening between 2002 and 2003. To ensure homogeneity, the cohort was restricted to adults aged 40–80 years as the screening program was not widely attended by young individuals and had a low response rate among those older than 80 years. The NHIS-HealS database includes three datasets: sociodemographic information, diagnostic information obtained through the 10th revision of the International Classification of Disease-10 (ICD-10) codes, and National Health Screening data [19]. The National Health check-ups included regular blood tests, chest radiographs, physical examinations, and medical history questionnaires. Statistics Korea provides data on deaths, including date and cause, through individual linkages using unique personal identification numbers [19, 20]. The Institutional Review Board of the Yonsei University Health System granted approval for this study (4-2016-0179), and the board waived the requirement for obtaining informed consent, and the study was conducted in accordance with the Declaration of Helsinki (1989) by the World Medical Association.

### 2.2 Study Cohort

**Fig. 1.**
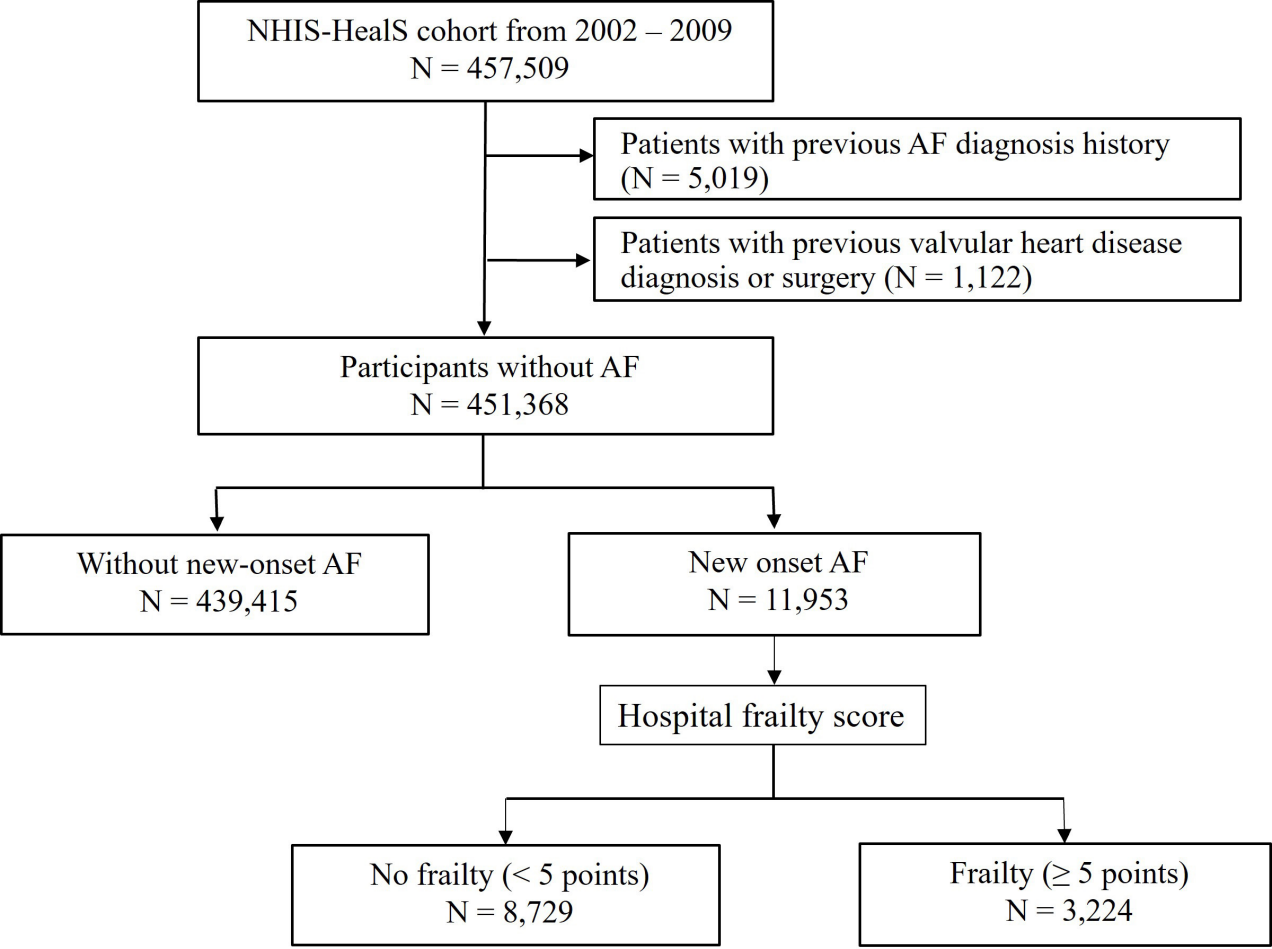
**Flow diagram of the study**. AF, atrial fibrillation; NHIS-HealS, Korea National Health Insurance Service-Health Screening.

This study included adults aged 40–80 years who underwent National Health check-ups between 2002 and 2009 (n = 457,509) from the NHIS-HealS cohort [19, 20]. The present study excluded patients with the following to reduce confounding factors: (i) previous AF diagnosis history (n = 5019), and (ii) valvular heart diseases including mitral valve stenosis and individuals with prosthetic valves (ICD-10 code: I050, I052, I342) (n = 1122). This study included 451,368 participants without AF (Fig. 1).

Information on the comorbidities in the NHIS-HealS data is provided in **Supplementary Table 1** and has been validated in previous studies [1, 2, 3, 15, 19, 20, 21, 22]. The ICD-10 codes are used in the NHIS-HealS data to define the presence of comorbidities at baseline. To ensure the accuracy of the dataset, we operationalized newly diagnosed AF as the initial occurrence documented on 2 or more separate days during outpatient hospital visits, or as the initial hospitalization with confirmed identification of AF using the ICD-10 code (I48). The positive predictive value of this dataset was 94.1% [19]. The Hospital Frailty Risk Score for each patient was calculated retrospectively, considering all available ICD-10 diagnosis codes recorded before the index date [23]. This score includes 109 ICD-10 codes related to frailty (**Supplementary Table 2**). Each code was assigned a value proportional to the degree of frailty. A score of at least five points was considered indicative of frailty [23]. We classified patients who were on OAC therapy for three months or longer during the follow-up period into the OAC group. The usage rate of OAC was 25.3% in this study population, which is consistent with other studies conducted in Korea during a similar time frame [24, 25, 26].

### 2.3 Follow-Up and Clinical Outcomes

The primary outcome assessed in the study was death from any cause. Death registration, based on death certificates, was performed by the National Population Registry of the Korea National Statistical Office [3, 15]. The secondary outcomes were cardiovascular death, ischemic stroke/transient ischemic attack, major bleeding defined based on the 2005 International Society on Thrombosis and Hemostasis criteria [27], and heart failure admission. Cardiovascular death was defined as death from cardiovascular disease, based on death certificate registration (**Supplementary Table 3**). Information on the outcomes of interest in the NHIS-HealS data is provided in **Supplemental Table 3** and has been confirmed in previous studies [3, 15].

### 2.4 Statistical Analysis

Descriptive data were expressed as mean ± standard deviation (SD) or median [interquartile range] for continuous variables and number (proportions) for categorical variables. Student’s *t*-test was used to compare continuous variables, whereas the chi-square and Fisher’s exact tests were used for categorical variables.

Logistic regression analysis was used to investigate the association between medication use and frailty. To include a variable in the multivariate model, the variable was required to meet a univariate significance level of 0.05. Additionally, for inclusion in the model, a variable had to meet a multivariate significance level of 0.05. The results were presented as odds ratios (OR) with corresponding 95% confidence intervals (CI). Cox proportional hazards regression models were used to assess the association between frailty and clinical outcomes. Similarly, a univariate significance level of 0.05 was required to allow a variable into the multivariate model, and a multivariate significance level of 0.05 was required for a variable to remain in the model. The results were adjusted for age, sex, and comorbidities and reported as hazard ratios (HR) with 95% CIs.

We independently calculated the annual incidence rates of the primary and secondary clinical outcomes in patients with and without frailty. The number of events was divided by the exposure period, measured in patient years (PYs), and the results were expressed as the number of events per 100 PYs. The *p*-interaction was employed to calculate the difference between the two annual event rates and to determine the associated *p*-value. Finally, survival analyses were conducted using Kaplan–Meier estimates to compare event-free survival distributions between the groups. The log-rank test was used to assess the significance of the differences between distributions. Two-sided *p*-values < 0.05 were considered statistically significant. Statistical analyses were conducted using the R version 4.1.2 (www.R-project.org; R Foundation for Statistical Computing, Vienna, Austria).

## 3. Results

### 3.1 Baseline Characteristics

Among 451,368 participants without AF, 11,953 (2.6%) incident AF cases occurred during a follow-up duration of 7.2 ± 1.5 years. Compared with controls, patients with new-onset AF had a higher prevalence of frailty, even when enrolled in the cohort (4.8 vs. 2.4, *p* < 0.001) (**Supplementary Table 4**).

In all patients with AF (median age, 67 [interquartile range, 59.5–74.5] years; 7200 [60.2%] males), the prevalence of frailty was increased to 26.9% at the time of AF diagnosis. Table 1 presents the demographic and clinical characteristics of the patients categorized according to the presence or absence of frailty when they were diagnosed with AF. Patients with frailty were aged and included more females compared to those without frailty. Compared to patients without frailty, the prevalence of several comorbidities was higher in patients with frailty, similar to the Charlson Comorbidity Index, Hospital frailty risk score, ${\mathrm{CHA}}_{2}$${\mathrm{DS}}_{2}$-VASc and HAS-BLED scores. However, the incidence of polypharmacy was lower in the patients with frailty than in those without.

**Table 1.** **Clinical characteristics of AF population according to frailty when AF diagnosed**.

		No frailty (n = 8729)	Frailty (n = 3224)	*p*-value
Age, years		66.0 [57.0; 72.0]	72.0 [65.0; 78.0]	<0.001
	Age 65–75	1514 (17.3)	1251 (38.8)	<0.001
	Age >75	2859 (32.8)	1109 (34.4)	<0.001
Male		5436 (62.3)	1764 (54.7)	<0.001
Body mass index		24.3 [22.3; 26.3]	23.7 [21.7; 25.8]	<0.001
Systolic blood pressure		128.0 [117.0; 138.0]	130.0 [119.0; 140.0]	<0.001
Diastolic blood pressure		80.0 [70.0; 85.0]	80.0 [70.0; 85.0]	0.532
Hospital frailty risk score		0.0 [0.0; 2.0]	9.2 [6.7; 13.8]	<0.001
${\mathrm{CHA}}_{2}$${\mathrm{DS}}_{2}$-VASc score		2.0 [1.0; 4.0]	4.0 [3.0; 6.0]	<0.001
HAS-BLED score		2.0 [1.0; 3.0]	2.0 [2.0; 3.0]	<0.001
Charlson comorbidity index		2.0 [1.0; 4.0]	5.0 [3.0; 8.0]	<0.001
Polypharmacy		2385 (27.3)	708 (22.0)	<0.001
Smoking group				0.052
	Ex-smoker	676 (25.3)	183 (21.8)	
	Current-smoker	362 (13.5)	104 (12.4)	
Alcohol group				<0.001
	Social-alcoholics	7362 (84.3)	3012 (93.4)	
	Heavy-alcoholics	1367 (15.7)	212 (6.6)	
Heart failure		1761 (20.2)	1085 (33.7)	<0.001
Hypertension		5127 (58.7)	2412 (74.8)	<0.001
Diabetes mellitus		1570 (18.0)	1145 (35.5)	<0.001
Ischemic stroke or TIA		1254 (14.4)	1328 (41.2)	<0.001
Previous MI		463 (5.3)	433 (13.4)	<0.001
Vascular disease		1108 (12.7)	769 (23.9)	<0.001
Major bleeding		861 (9.9)	638 (19.8)	<0.001
ESRD or CKD		194 (2.2)	329 (10.2)	<0.001
COPD		1010 (11.6)	834 (25.9)	<0.001
Malignancy		1909 (21.9)	755 (23.4)	0.075

Data are expressed as mean [interquartile range] (percent). Index date was the date of AF diagnosis. AF, atrial fibrillation; CKD, chronic kidney disease; COPD, chronic obstructive pulmonary disease; ESRD, end-stage renal disease; MI, myocardial infarction; TIA, transient ischemic attack.

### 3.2 Frailty and OAC Prescription

The baseline prescription rate of OAC before the AF diagnosis was significantly higher in patients with AF and frailty than in those without frailty (5.1% vs. 3.4%, *p* < 0.001). The prescription rate of OAC after the diagnosis of new-onset AF was lower in patients with AF and frailty than in those without frailty (20.9% vs. 26.2%, *p* < 0.001). The use of anti-platelet agents was higher before AF diagnosis (49.8% vs. 36.5%, *p* < 0.001) and lower after AF diagnosis in patients with AF and frailty than in those without frailty (48.7% vs. 62.7%, *p* < 0.001). A consistent trend was observed in the AF population at high risk of ischemic stroke (${\mathrm{CHA}}_{2}$${\mathrm{DS}}_{2}$-VASc ≥2 male or ≥3 female) (Table 2).

**Table 2.** **OAC and anti-platelet agent prescription rate according to frailty in AF population**.

Overall AF population				
		No frailty (n = 8729)	Frailty (n = 3224)	*p*-value
OAC				
	before AF	299 (3.4)	164 (5.1)	<0.001
	after AF	2285 (26.2)	674 (20.9)	<0.001
Anti-platelet agent				
	before AF	3185 (36.5)	1607 (49.8)	<0.001
	after AF	5472 (62.7)	1569 (48.7)	<0.001
AF population with high stroke risk (${\mathrm{CHA}}_{2}$${\mathrm{DS}}_{2}$-VASc ≥2 male or ≥3 female)				
		No frailty (n = 5363)	Frailty (n = 2791)	*p*-value
OAC				
	before AF	242 (4.5)	156 (5.6)	0.037
	after AF	1527 (28.5)	599 (21.5)	<0.001
Anti-platelet agent				
	before AF	2745 (51.2)	1562 (56.0)	<0.001
	after AF	3689 (68.8)	1428 (51.2)	<0.001

Data are expressed as number (percent). AF, atrial fibrillation; OAC, oral anti-coagulants.

The factors associated with OAC prescriptions are presented in Table 3. Before AF, frailty was associated with a higher OAC prescription (OR 1.37, 95% CI 1.11–1.70, *p* = 0.003). However, frailty was negatively associated with OAC prescription after AF diagnosis (OR 0.67, 95% CI 0.60–0.75, *p* < 0.001) (Table 3).

**Table 3.** **Coefficients associated with OAC prescription in AF population**.

	Before AF				After AF			
	Univariate analysis		Multivariate analysis		Univariate analysis		Multivariate analysis	
	OR (95% CI)	*p*-value	OR (95% CI)	*p*-value	OR (95% CI)	*p*-value	OR (95% CI)	*p*-value
Frailty	1.51 (1.24–1.84)	<0.001	1.37 (1.11–1.70)	0.003	0.75 (0.68–0.82)	<0.001	0.67 (0.60–0.75)	<0.001
Male	0.90 (0.74–1.08)	0.249	-	-	1.16 (1.06–1.26)	<0.001	1.11 (1.00–1.22)	0.045
Heart failure	2.71 (2.25–3.27)	<0.001	2.09 (1.71–2.56)	<0.001	1.41 (1.28–1.55)	<0.001	1.51 (1.37–1.66)	<0.001
Hypertension	2.54 (2.01–3.21)	<0.001	1.60 (1.24–2.08)	<0.001	1.13 (1.04–1.23)	0.005	-	-
Diabetes	1.19 (0.96–1.47)	0.118	-	-	0.88 (0.80–0.97)	0.013	0.88 (0.79–0.97)	0.012
Ischemic stroke	2.65 (2.19–3.20)	<0.001	2.11 (1.72–2.57)	<0.001	1.32 (1.20–1.46)	<0.001	1.49 (1.34–1.66)	<0.001
Previous MI	2.69 (2.09–3.46)	<0.001	1.72 (1.32–2.24)	<0.001	1.08 (0.93–1.26)	0.327	-	-
Vascular disease	2.23 (1.81–2.74)	<0.001	-	-	1.07 (0.95–1.19)	0.259	-	-
Osteoporosis	0.96 (0.78–1.18)	0.722	-	-	0.80 (0.72–0.87)	<0.001	0.82 (0.73–0.91)	<0.001
Dyslipidemia	1.80 (1.47–2.20)	<0.001	1.28 (1.04–1.59)	0.022	1.08 (0.99–1.17)	0.075	-	-

AF, atrial fibrillation; CI, confidence interval; MI, myocardial infarction; OAC, oral anti-coagulants; OR, odds ratio.

### 3.3 Frailty and Adverse Events

**Fig. 2.**
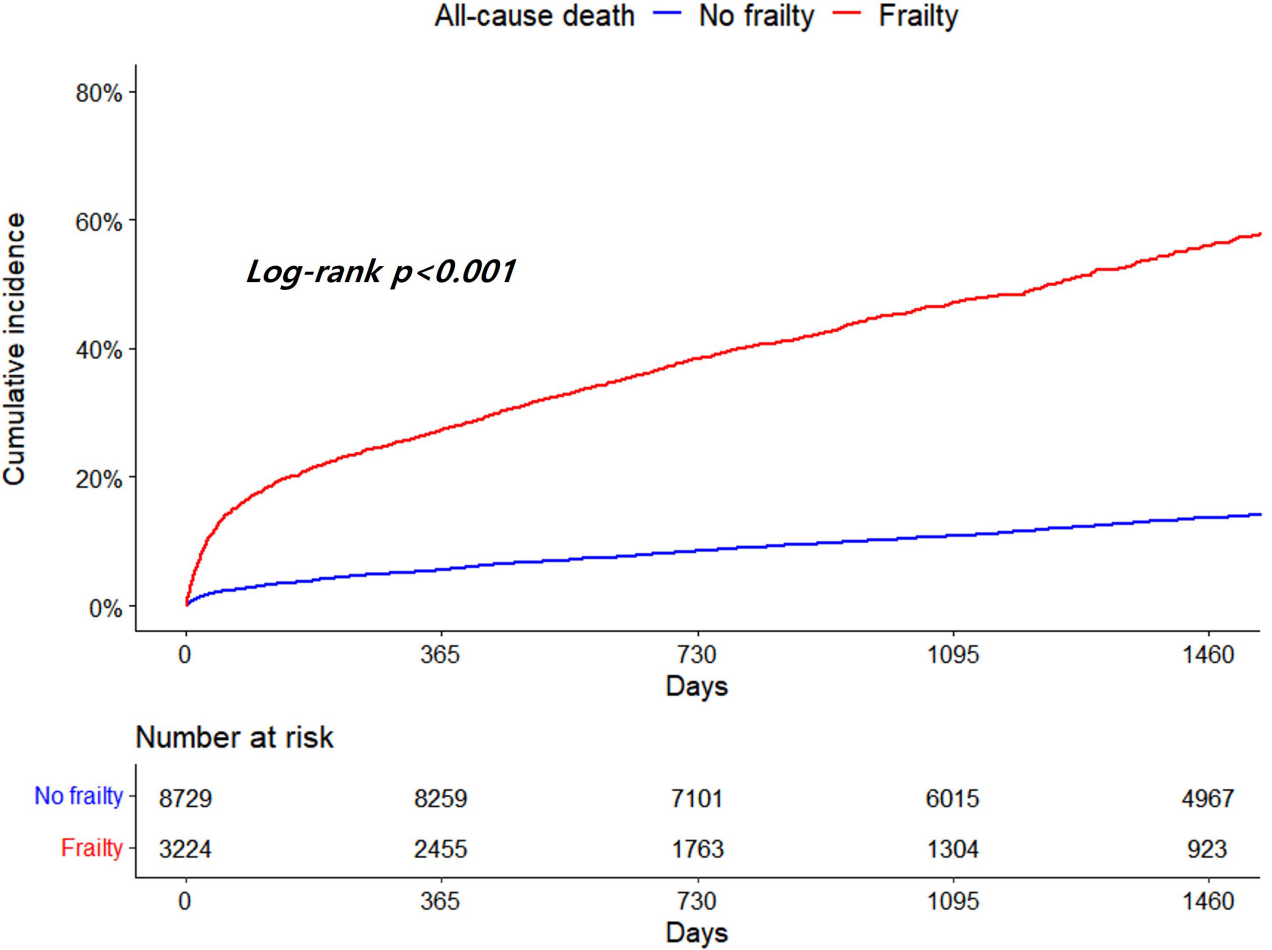
**Kaplan-Meier curves for all-cause death according to frailty**.

Patients with frailty demonstrated significantly higher all-cause death and risk of that than those without frailty (14.61 vs. 3.40 per 100 PYs, adjusted HR 2.88, 95% CI 2.65–3.14, *p* < 0.001) (Table 4). The cumulative incidence of all-cause death is depicted in Fig. 2 (log-rank *p* < 0.001).

**Table 4.** **Incidence and hazard ratio for all-cause death and other clinical outcomes according to frailty**.

		Incidence rate (/100 person-years)		Adjusted HR ${\mathrm{(95\% CI)}}^{†}$	*p*-value
		No frailty (n = 8729)	Frailty (n = 3224)		
Primary outcome					
	All-cause death	3.40	14.61	2.88 (2.65–3.14)	<0.001
Secondary outcome					
	Cardiovascular death	1.14	4.95	2.42 (2.10–2.80)	<0.001
	Ischemic stroke	2.49	9.79	2.25 (2.02–2.51)	<0.001
	Major bleeding	2.27	8.02	2.44 (2.17–2.73)	<0.001
	Heart failure admission	1.22	2.92	1.29 (1.09–1.52)	0.004

^†^Adjusted for age, sex, and a medical history that includes heart failure, hypertension, diabetes, ischemic stroke, previous myocardial infarction, vascular disease, osteoporosis, and dyslipidemia. CI, confidence interval; HR, hazard ratio.

**Fig. 3.**
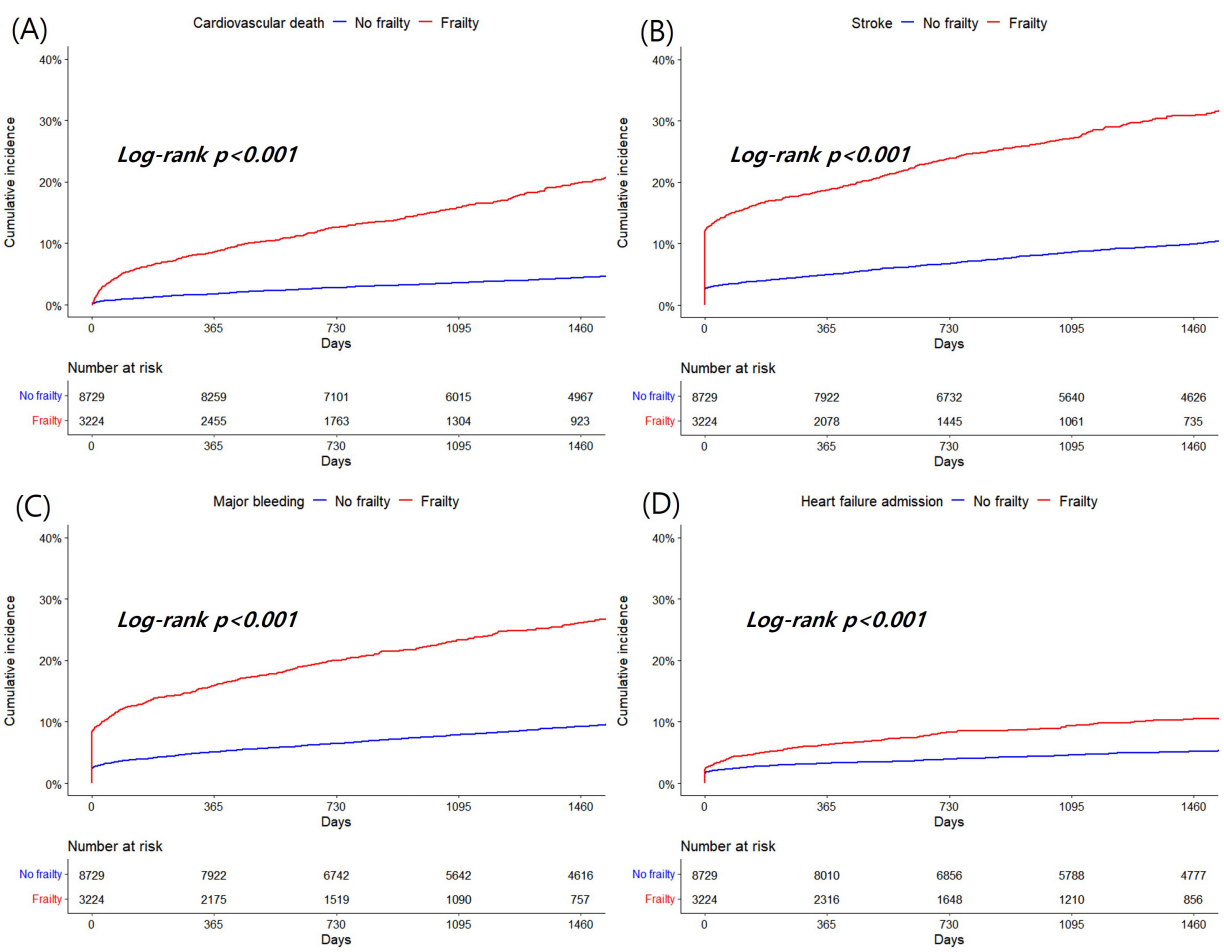
**Kaplan-Meier curves for cardiovascular death (A), ischemic stroke (B), major bleeding (C) and heart failure admission (D) according to frailty**.

Among secondary outcomes, patients with frailty displayed significantly higher incidence and risk of cardiovascular death (4.95 vs. 1.14 per 100 PYs, adjusted HR 2.42, 95% CI 2.10–2.80, *p* < 0.001), ischemic stroke (9.79 vs. 2.49 per 100 PYs, adjusted HR 2.25, 95% CI 2.02–2.51, *p* < 0.001), major bleeding (8.02 vs. 2.27 per 100 PYs, adjusted HR 2.44, 95% CI 2.17–2.73, *p* < 0.001) and heart failure admission (2.92 vs. 1.22 per 100 PYs, adjusted HR 1.29, 95% CI 1.09–1.52, *p* = 0.004) compared to patients without frailty (Table 4). Fig. 3 displays the cumulative incidences of cardiovascular death, ischemic stroke, major bleeding, and heart failure admission (all log-rank *p* < 0.001).

### 3.4 OAC, Frailty and Adverse Events

Patients with frailty also demonstrated a significantly higher risk of all-cause death (no OAC group, adjusted HR 3.04, 95% CI 2.78–3.34; OAC group, adjusted HR 1.93, 95% CI 1.56–2.38) and secondary outcomes compared to those without frailty. The cumulative incidence of all-cause death and secondary outcomes was significantly higher in patients with frailty than in those without frailty, regardless of OAC use (**Supplementary Figs. 1**,**2**,**3**).

However, the risk of all-cause death due to frailty was significantly lower in the OAC group than in the no OAC group (adjusted HR 1.93 vs. 3.04, *p*-interaction <0.001). The risks of cardiovascular death, ischemic stroke, and heart failure admission due to frailty were also lower in the OAC group than in the no OAC group (Table 5).

**Table 5.** **Incidence and hazard ratio for all-cause death and other clinical outcomes according to frailty and OAC use**.

		Incidence rate (/100 person-years)		Adjusted HR ${\mathrm{(95\% CI)}}^{†}$	*p*-value	*p*-interaction
		No frailty	Frailty			
Primary outcome						
All-cause death						<0.001
	No OAC (n = 8925)	3.64	17.62	3.04 (2.78–3.34)	<0.001	
	OAC (n = 3028)	2.77	6.86	1.93 (1.56–2.38)	<0.001	
Secondary outcome						
Cardiovascular death						0.007
	No OAC (n = 8925)	1.10	5.50	2.53 (2.14–2.98)	<0.001	
	OAC (n = 3028)	1.24	3.54	1.99 (1.47–2.69)	<0.001	
Ischemic stroke						<0.001
	No OAC (n = 8925)	1.62	8.05	2.57 (2.22–2.96)	<0.001	
	OAC (n = 3028)	4.95	15.40	2.26 (1.91–2.68)	<0.001	
Major bleeding						0.082
	No OAC (n = 8925)	2.14	8.35	2.50 (2.18–2.85)	<0.001	
	OAC (n = 3028)	2.60	7.13	2.22 (1.77–2.78)	<0.001	
Heart failure admission						0.004
	No OAC (n = 8925)	0.91	2.87	1.47 (1.19–1.82)	<0.001	
	OAC (n = 3028)	2.01	3.19	1.08 (0.81–1.45)	0.601	

^†^Adjusted for age, sex, and a medical history that includes heart failure, hypertension, diabetes, ischemic stroke, previous myocardial infarction, vascular disease, osteoporosis, and dyslipidemia. CI, confidence interval; HR, hazard ratio; OAC, oral anti-coagulants.

## 4. Discussion

The primary finding of this study was that frailty was associated with higher risks of all-cause death and poorer clinical outcomes in patients with AF compared to those without frailty. Secondly, frailty was more prevalent and was negatively associated with OAC use in patients with AF. Finally, OAC use was associated with lower risks related to frailty for all-cause death and poorer clinical outcomes in patients with AF. This finding suggests that appropriate OAC use is important for improving the clinical outcomes of patients with frailty.

### 4.1 High Frailty in Patients Affected by Atrial Fibrillation

In older adults, AF may serve as an indicator of frailty and may be associated with a decline in the ability to independently perform daily activities [9, 10]. In this study, when participants were enrolled in the health examination cohort, the prevalence of frailty was only 2.4% in those without future AF, and 4.8% in those with future AF. However, among participants with AF, the prevalence of frailty increased by 5.6 times to 26.9%. Although a direct comparison of the prevalence of frailty is impossible because of the difference in the definition of AF, it has been reported that AF increases the risk of frailty, with individuals having a four-fold higher likelihood of being classified as frail than those without AF [11]. The dramatic increase in frailty observed in the AF population might be related to old age and high comorbidities. The incidence of AF and frailty increases progressively with age, ranging from 0.1% in patients aged <55 years to >9% in octogenarians [28]. Although AF can be independently associated with frailty, it is important to note that aging has the most significant impact on the relationship between AF and frailty [29]. In this study, the Charlson Comorbidity Index was twice as high in the frail group than in the non-frail group. Notably, the frail group received fewer medications and had a lower prevalence of polypharmacy compared to the non-frail group. Reducing polypharmacy is the right strategy to prevent and manage frailty [30]. However, conducting a risk-benefit assessment is essential to avoid excluding important medications during this procedure.

### 4.2 OAC of AF Patients with Frailty

Many studies have demonstrated that frail patients with AF are less likely to receive anticoagulation therapy despite an increasing incidence of ischemic stroke than non-frail patients [31, 32]. In this study, frailty was negatively associated with OAC and anti-platelet treatment. Kim *et al*. [16] reported that they did not observe any significant increase in the risk of bleeding outcomes. However, protective associations of OAC treatment with low risks of ischemic stroke and mortality were consistently observed in frail patients with AF [16]. This study consistently demonstrated that the increased risk of all-cause death and major adverse cardiovascular events associated with frailty was reduced by OAC in patients with AF. Interestingly, we observed a higher incidence of ischemic stroke in the OAC group than that in the non-OAC group. However, the risk of ischemic stroke attributed to frailty was lower in patients with OAC prescriptions than in those without OAC. Several patients were simultaneously diagnosed with AF and ischemic stroke. Therefore, the present study yielded the aforementioned results. Additionally, OAC administration did not increase the risk of major bleeding due to frailty. However, caution is needed when interpreting this finding because patients with a high bleeding risk may not have been prescribed OACs from the beginning. In summary, OAC use may play a crucial role in improving outcomes in patients with AF and frailty. The appropriate prescription of anticoagulation therapy may seem ideal, but many factors are present that should be considered, such as the physician’s experience. Moreover, whether a single episode of AF should be treated in every patient or if subcategories should be considered, especially in light of new tools that allow rhythm evaluation, which could be of significant benefit to frail older patients, remains unclear. Furthermore, there are many novel risk stratification tools available that are more suitable than the conventional ${\mathrm{CHA}}_{2}$${\mathrm{DS}}_{2}$-VASc and HAS-BLED scores [33].

### 4.3 High Adverse Events in Patients with Frailty

Frailty may serve as an indicator of health conditions among patients with AF, as it identifies patients with an increased risk profile of multiple comorbidities and can aid in identifying those who are frail. Additionally, frailty is common among patients with AF and contributes to poor clinical outcomes [15, 16, 17]. In this study, frailty increased the risk of death from any cause by approximately three times and increased other clinical outcomes by approximately 1.5–2.5 times when compared to individuals without frailty. Paying special attention to and focusing on the follow-up of frail older patients is important. Additionally, evaluating the risk-benefit ratio of each treatment is necessary to avoid omitting essential medications, such as OAC.

Considering the high healthcare burden associated with AF and frailty, integrated AF management should be implemented to improve outcomes [15]. In previous studies, the management of AF was effectively addressed by adopting an integrated and holistic Atrial fibrillation Better Care (ABC) pathway, avoiding ischemic stroke by using OAC therapy, better symptom management, and cardiovascular risk factors and comorbidities optimization [34]. Consequently, optimal medical therapy is associated with improved outcomes in patients with AF and a high risk of frailty. Integrated management in patients with AF and frailty is consistent with previous studies that have demonstrated the benefits of integrated and holistic management using the ABC pathway [35], OAC, and early rhythm control therapy [17].

### 4.4 Limitations

Our study was a large-scale Korean NHIS-HealS cohort of approximately 500,000 individuals who underwent health check-ups. Nonetheless, this study has certain limitations. First, investigations using administrative databases may be susceptible to errors arising from coding inaccuracies. To minimize this, we used definitions previously validated for the Korean NHIS-HealS cohort [1, 2, 3, 15, 19, 20, 21, 22]. There were differences in the follow-up duration among the groups. These differences may be attributed to variations in mortality rates within each group, which could influence the results and serve as a confounding factor. We could not separately analyze initial, paroxysmal, persistent, and permanent AF or atrial flutter. Moreover, we are unsure whether any asymptomatic or unrecognized AF existed in patients before enrollment in the study, given the characteristics of the epidemiological cohort dataset. This factor may have influenced the results. Additionally, we did not distinguish between vitamin K antagonists and direct oral anticoagulants in the OAC prescriptions. Vitamin K antagonists have a narrow therapeutic range and no information is available on the attainment of appropriate INR control in patients using these agents. This factor could have affected the efficacy and safety outcomes of this study. The doses and types of direct oral anticoagulants administered were indistinguishable. Furthermore, there may be issues related to the accuracy of OAC adherence and ambiguity in assessing the effects of OAC. The variability in the timing of OAC initiation could potentially impact the study results. We also analyzed a specific population of patients with new-onset AF, rather than the entire Korean NHIS cohort. These issues should be investigated further in future studies.

## 5. Conclusions

In this nationwide cohort, we demonstrated that frailty was highly prevalent in the population with AF, was negatively associated with the use of OAC, and was a predictor of poor prognosis due to its association with death, thromboembolic events, bleeding, and admission for heart failure. However, OAC use was associated with lower risks related to frailty for all-cause death and major adverse cardiovascular events in patients with AF. This study supports the use of OAC and optimal integrated management of patients with AF and frailty.

## Data Availability

The dataset analyzed during this study are available from the corresponding author on reasonable request.

## Supplementary Material

Supplementary material associated with this article can be found, in the online version, at https://doi.org/10.31083/j.rcm2502052.
