# Supplementary material for: All-Cause Death and Major Adverse Events in Atrial Fibrillation with Frailty: Observations from the Korea National Health Insurance Service Data
Source: Rev Cardiovasc Med. 2024 Jan 30;25(2):52. doi: 10.31083/j.rcm2502052 (PMC11263154; doi:10.31083/j.rcm2502052)

Supplementary Materials

Supplementary Table 1. Definitions and codes used for defining medical conditions, comorbidities, and drug treatments and procedures for atrial fibrillation.

	Definitions	Codes or conditions
**Medical conditions**		
Atrial fibrillation	Defined from diagnosis*	I48
Heart failure	Defined from diagnosis*	ICD-10: I11.0, I50, I97.1
Heart failure admission history	Defined from principal or first secondary admission diagnoses of heart failure	ICD-10: I11.0, I50, I97.1
Hypertension	Defined if fulfilling both diagnosis* and treatment within 90 days prior to the first recorded prescription or procedure for rhythm or rate control	ICD-10: I10, I11, I12, I13, I15<br>Treatment: prescription for at least one of all kinds of antihypertensive medication
Diabetes mellitus	Defined if fulfilling both diagnosis* and treatment within 90 days prior to the first recorded prescription or procedure for rhythm or rate control	ICD-10: E10, E11, E12, E13, E14<br>Treatment: prescription for at least one of all kinds of oral antidiabetics or insulin
Dyslipidemia	Defined from diagnosis*	ICD-10: E78
Ischemic stroke	Defined from diagnosis*	ICD-10: I63, I64
Transient ischemic attack	Defined from diagnosis*	ICD-10: G45
Major bleeding	Defined from diagnosis*	ICD-10: I60, I61, I62<br>K25, K26, K27, K28<br>(subcodes 0-2 and 4-6 only)<br>K92.0, K92.1, K92.2, K62.5, I85.0, I98.3, D62
Myocardial infarction	Defined from diagnosis*	ICD-10: I21, I22, I25.2
Vascular disease	Defined from diagnosis*	ICD-10: I70.0, I70.1, I70.2, I70.8, I70.9
Valvular heart disease	Defined from diagnoses* mitral stenosis or claims for heart valve surgery	ICD-10: I05.0, I05.2, I34.2, Z95.2-4<br>Claim for valve replacement or valvuloplasty: O1781, O1782, O1783, O1791, O1792, O1793, O1797, O1794, O1795, O1796, O1798
Chronic kidney disease	Defined from eGFR or diagnosis* (if laboratory value was not available, diagnosis code was used)	eGFR <60mL/min per 1.73 m^2<br>ICD-10: N18, N19
ESRD	Defined from national registry for severe illness	Patients with ESRD undergoing chronic dialysis or received a kidney transplant.
Malignancy	Defined from diagnoses* of cancer (non-benign)	ICD-10: C00-C97
Chronic obstructive pulmonary disease	Defined if fulfilling both diagnosis* and treatment within 90 days prior to the first recorded prescription or procedure for rhythm or rate control	ICD-10: J42, J43(except J43.0), J44<br>Treatment: SABA, SAMA, LABA, LAMA, ICS, ICS+LABA, or methylxanthine (>1 months).
Chronic liver disease	Defined from diagnosis* of chronic liver disease, cirrhosis, and hepatitis	ICD-10: B18, K70, K71, K72, K73, K74, K76.1
Osteoporosis	Defined from diagnosis*	ICD-10: M80, M81, M82 (except M82.0)
**Drug treatment for atrial fibrillation (available in South Korea)**		
Anti-arrhythmic drug		

Class Ic	flecainide, pilsicainide, propafenone
Class III	amiodarone, dronedarone, sotalol
Rate control drugs	
Beta-blocker	atenolol, bisoprolol, carvedilol, metoprolol, nebivolol, propranolol, labetalol
Calcium channel blocker	diltiazem, verapamil
Cardiac glycosides	digoxin
OACs	
Warfarin	warfarin
NOACs	apixaban, dabigatran, edoxaban, rivaroxaban
Antiplatelet agents	
Aspirin	aspirin
P2Y12 inhibitors	clopidogrel, prasugrel, ticagrelor

*For greater accuracy, either one diagnosis during hospitalization or more than twice at outpatient clinics was requited for the diagnosis.

eGFR, estimated glomerular filtration rate; ESRD, end-stage renal disease; ICD-10, International Classification of Diseases 10th Revision; ICS, inhaled corticosteroids; LABA, long-acting bronchodilator combinations; LAMA, long-acting antimuscarinic antagonists; NOACs, non-vitamin K-antagonist oral anticoagulants; OACs, oral anticoagulants; PPV, positive predictive value; SABA, short-acting bronchodilator combinations; SAMA, short-acting antimuscarinic antagonists.

Supplementary Table 2. List of 109 variables contributing to calculating the Hospital Frailty Risk Score.

ICD-10 Description	ICD-10 code	Points
Dementia in Alzheimer's disease	F00	7.1
Hemiplegia	G81	4.4
Alzheimer's disease	G30	4
Sequelae of cerebrovascular disease (secondary codes)	I69	3.7
Other symptoms and signs involving the nervous and musculoskeletal systems (R29.6 Tendency to fall)	R29	3.6
Other disorders of urinary system (includes urinary tract infection and urinary incontinence)	N39	3.2
Superficial injury of head	S00	3.2
Delirium, not induced by alcohol and other psychoactive substances	F05	3.2
Unspecified fall	W19	3.2
Unspecified hematuria	R31	3
Other bacterial agents as the cause of diseases classified to other chapters (secondary code)	B96	2.9
Other symptoms and signs involving cognitive functions and awareness	R41	2.7
Other cerebrovascular diseases	I67	2.6
Convulsions, not elsewhere classified	R56	2.6
Abnormalities of gait and mobility	R26	2.6
Somnolence, stupor and coma	R40	2.5
Intracranial injury	S06	2.4
Complications of genitourinary prosthetic devices, implants and grafts	T83	2.4
Other disorders of fluid, electrolyte and acid base balance	E87	2.3
Other joint disorders, not elsewhere classified	M25	2.3
Volume depletion	E86	2.3
Fracture of shoulder and upper arm	S42	2.3
Senility	R54	2.2
Unspecified dementia	F03	2.1
Care involving use of rehabilitation procedures	Z50	2.1
Other fall on same level	W18	2.1
Cellulitis	L03	2
Vascular dementia	F01	2
Superficial injury of lower leg	S80	2
Problems related to medical facilities and other health care	Z75	2
Deficiency of other B group vitamins	E53	1.9
Blindness and low vision	H54	1.9
Other functional intestinal disorders	K59	1.8
Fracture of rib(s), sternum and thoracic spine	S22	1.8
Syncope and collapse	R55	1.8
Acute renal failure	N17	1.8
Parkinson's disease	G20	1.8
Problems related to social environment	Z60	1.8
Decubitus ulcer	L89	1.7
Carrier of infectious disease	Z22	1.7
Streptococcus and staphylococcus as the cause of diseases classified to other chapters	B95	1.7
Other septicemia	A41	1.6
Duodenal ulcer	K26	1.6
Hypotension	I95	1.6
Unspecified renal failure	N19	1.6
Ulcer of lower limb, not elsewhere classified	L97	1.6
Other symptoms and signs involving general sensations and perceptions	R44	1.6

Epilepsy	G40	1.5
Other arthrosis	M19	1.5
Respiratory failure, not elsewhere classified	J96	1.5
Personal history of other diseases and conditions	Z87	1.5
Exposure to unspecified factor	X59	1.5
Osteoporosis without pathological fracture	M81	1.4
Abnormal results of function studies	R94	1.4
Fracture of lumbar spine and pelvis	S32	1.4
Chronic renal failure	N18	1.4
Fracture of femur	S72	1.4
Other disorders of pancreatic internal secretion	E16	1.4
Other disorders of kidney and ureter, not elsewhere classified	N28	1.3
Retention of urine	R33	1.3
Unknown and unspecified causes of morbidity	R69	1.3
Transient cerebral ischemic attacks and related syndromes	G45	1.2
Other degenerative diseases of nervous system, not elsewhere classified	G31	1.2
Unspecified urinary incontinence	R32	1.2
Symptoms and signs involving emotional state	R45	1.2
Other and unspecified injuries of head	S09	1.2
Nosocomial condition	Y95	1.2
Pneumonia, organism unspecified	J18	1.1
Diarrhea and gastroenteritis of presumed infectious origin	A09	1.1
Other soft tissue disorders, not elsewhere classified	M79	1.1
Open wound of head	S01	1.1
Other bacterial intestinal infections	A04	1.1
Fall involving bed	W06	1.1
Problems related to care-provider dependency	Z74	1.1
Speech disturbances, not elsewhere classified	R47	1
Pneumonitis due to solids and liquids	J69	1
Artificial opening status	Z93	1
Vitamin D deficiency	E55	1
Gangrene, not elsewhere classified	R02	1
Thyrotoxicosis [hyperthyroidism]	E05	0.9
Symptoms and signs concerning food and fluid intake	R63	0.9
Other hearing loss	H91	0.9
Scoliosis	M41	0.9
Fall on same level from slipping, tripping and stumbling	W01	0.9
Fall on and from stairs and steps	W10	0.9
Cerebral Infarction	I63	0.8
Other diseases of digestive system	K92	0.8
Dysphagia	R13	0.8
Osteoporosis with pathological fracture	M80	0.8
Agent resistant to penicillin and related antibiotics	U80	0.8
Dependence on enabling machines and devices	Z99	0.8
Abnormalities of heart beat	R00	0.7
Calculus of kidney and ureter	N20	0.7
Mental and behavioral disorders due to use of alcohol	F10	0.7
Unspecified acute lower respiratory infection	J22	0.7
Other medical procedures as the cause of abnormal reaction of the patient	Y84	0.7
Other abnormal findings of blood chemistry	R79	0.6
Problems related to life-management difficulty	Z73	0.6
Spinal stenosis (secondary code only)	M48	0.5
Depressive episode	F32	0.5
Open wound of forearm	S51	0.5

Personal history of risk-factors, not elsewhere classified	Z91	0.5
Other anemia	D64	0.4
Disorders of mineral metabolism	E83	0.4
Polyarthrosis	M15	0.4
Other local infections of skin and subcutaneous tissue	L08	0.4
Nausea and vomiting	R11	0.3
Other noninfective gastroenteritis and colitis	K52	0.3
Fever of unknown origin	R50	0.1

ICD-10, International Classification of Diseases-10th Revision.

Supplementary Table 3. Definitions and codes used for study outcomes.

Outcomes	Definitions	Codes or conditions	PPV
Ischemic stroke	Defined from admission diagnosis with concomitant imaging studies of the brain or related death	ICD-10: I63, I64	90.6%* (2347/2591)
Hospitalization owing to heart failure	Defined from principal or first secondary admission diagnoses of heart failure	ICD-10: I11.0, I50, I97.1	82.1%* (110/134)
Major bleeding	Defined from admission diagnosis with concomitant imaging studies of the brain or related death	ICD-10: I60, I61, I62 K25, K26, K27, K28 (subcodes 0-2 and 4-6 only) K92.0, K92.1, K92.2, K62.5, I85.0, I98.3, D62	-
Cardiovascular death		ICD-10: I05, I06, I07, I08, I09, I10, I11, I12, I13, I20, I21, I22, I23, I24, I25, I26, I27, I28, I30, I31, I32, I33, I34, I35, I36, I37, I38, I39, I40, I41, I42, I43, I44, I45, I46, I47, I48, I49, I50, I51, I60, I61, I62, I63, I64, I65, I66, I67, I68, I69, I70, I71, I72, I73, I74, I77, I80, I82	-

PPV was represented as % (number of true positive cases / number of examined cases).
*Validated in a study by Kim, D. et al. (Treatment timing and the effects of rhythm control strategy in patients with atrial fibrillation: nationwide cohort study. *BMJ* 2021;373:n991).
ICD-10, International Classification of Diseases-10th Revision; PPV, positive predictive value.

Supplementary Table 4. Characteristics of participants without and with new onset atrial fibrillation when enrolled the cohort.

	No AF (n=439,127)	New onset AF (n=12,241)	*p*-value
Age, year-old	54.0 [48.0;62.0]	63.0 [54.0;70.0]	<0.001
Age 65-75	15494 (3.5)	1284 (10.5)	<0.001
Age >75	65137 (14.8)	3857 (31.5)	<0.001
Male	236278 (53.8)	7342 (60.0)	<0.001
Body mass index	23.9 [22.0;25.8]	24.2 [22.2;26.2]	<0.001
Systolic blood pressure	125.0 [114.0;136.0]	130.0 [120.0;140.0]	<0.001
Diastolic blood pressure	80.0 [70.0;85.0]	80.0 [70.0;88.0]	<0.001
Hospital frailty risk score	0.45 ± 1.54	0.77 ± 2.09	<0.001
Hospital frailty risk categories			<0.001
low-risk (<5 points)	428406 (97.6)	11653 (95.2)	
Intermediate or high-risk (≥5 points)	10721 (2.4)	588 (4.8)	
CHA_2DS_2-VASc score	1.0 [0.0;2.0]	2.0 [1.0;3.0]	<0.001
HAS-BLED score	0.0 [0.0;1.0]	2.0 [1.0;2.0]	<0.001
Charlson comorbidity Index	1.0 [0.0;2.0]	2.0 [1.0;3.0]	<0.001
Polypharmacy	33648 (7.7)	4199 (34.3)	<0.001
Smoking group			0.001
Ex-smoker	38246 (9.2)	1160 (10.1)	
Current-smoker	77957 (18.7)	2049 (17.8)	
Alcohol group			0.048
Social-alcoholics	327065 (74.5)	9020 (73.7)	
Heavy-alcoholics	112062 (25.5)	3221 (26.3)	
Heart failure	108908 (24.8)	5411 (44.2)	<0.001
Hypertension	108908 (24.8)	5411 (44.2)	<0.001
Diabetes mellitus	35760 (8.1)	1623 (13.3)	<0.001
Ischemic stroke or TIA	20314 (4.6)	1201 (9.8)	<0.001
Previous MI	4425 (1.0)	385 (3.1)	<0.001
Vascular disease	12856 (2.9)	826 (6.7)	<0.001
Major bleeding	5241 (1.2)	235 (1.9)	<0.001
ESRD or CKD	3274 (0.7)	196 (1.6)	<0.001
COPD	12179 (2.8)	856 (7.0)	<0.001
Malignancy	29883 (6.8)	1323 (10.8)	<0.001

Data are expressed as mean [interquartile range] (percent), mean ± SD.

Index date was the date of enrollment.

AF = atrial fibrillation; CKD = chronic kidney disease; COPD = chronic obstructive pulmonary disease; ESRD = end-stage renal disease; MI = myocardial infarction; SD = standard deviation; TIA = transient ischemic attack.

Supplementary Figure 1. Kaplan-Meier curves for all-cause death according to frailty in patients without (A) and with OAC (B).

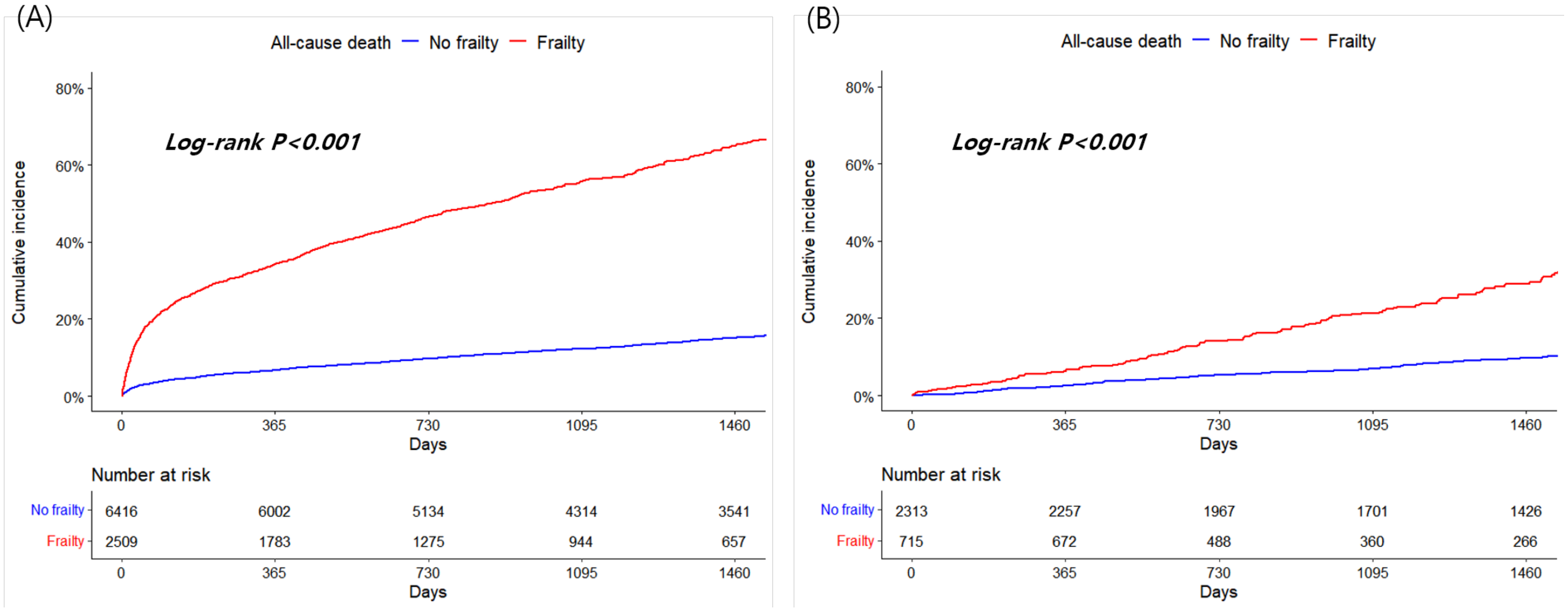

Supplementary Figure 2. Kaplan-Meier curves for cardiovascular death (A), stroke (B), major bleeding (C) and heart failure admission (D) according to frailty in patients without OAC.

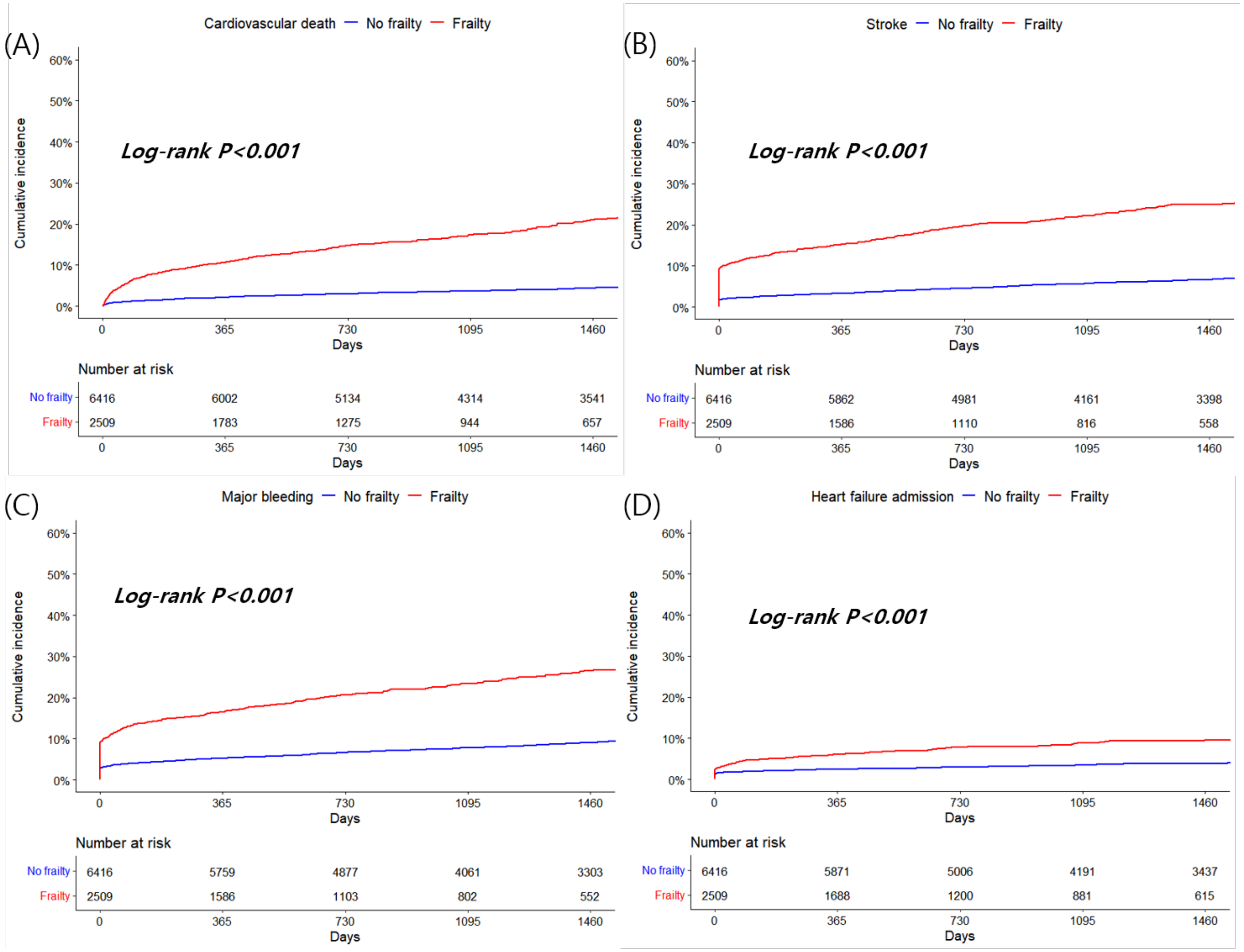

Supplementary Figure 3. Kaplan-Meier curves for cardiovascular death (A), stroke (B), major bleeding (C) and heart failure admission (D) according to frailty in patients with OAC.

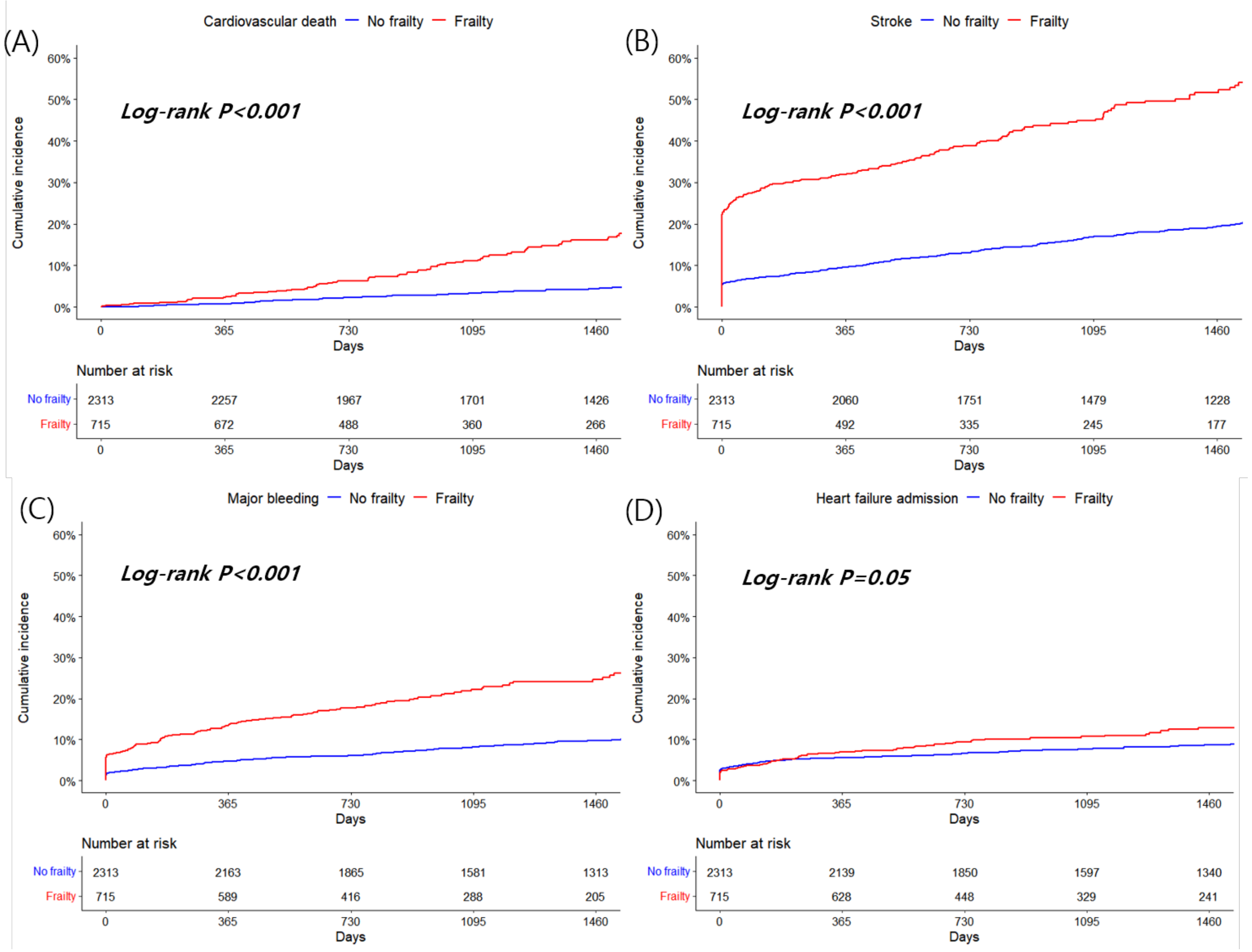

Supplement: Supplementary file 1 [file 2153-8174-25-2-052-s1.pdf]
